# Association between Cardiovascular Burden and Requirement of Intensive Care among Patients with Mild COVID-19

**DOI:** 10.1155/2020/9059562

**Published:** 2020-08-01

**Authors:** Shi Tai, Jianjun Tang, Bilian Yu, Liang Tang, Yang Wang, Huilin Zhang, Weihong Zhu, Kui Xiao, Chuan Wen, Chongqin Tan, Zhongbiao Jiang, Chuanhao Jiang, Li Zhu, Li Jiang, Qiming Liu, Xinqun Hu, Zhenfei Fang, Xuping Li, Jiaxing Sun, Zhaowei Zhu, Hui Yang, Tao Tu, Yichao Xiao, Mingxian Chen, Yuhu He, Xiangping Chai, Junmei Xu, Shenghua Zhou

**Affiliations:** ^1^Department of Cardiovascular Medicine, The Second Xiangya Hospital of Central South University, Changsha, China; ^2^National Emergency Medical Team, China; ^3^Department of Emergency Medicine, The Second Xiangya Hospital of Central South University, Changsha, China; ^4^Department of Anesthesiology, The Second Xiangya Hospital of Central South University, Changsha, China

## Abstract

**Background:**

Information regarding the impact of cardiovascular (CV) conditions on disease progression among patients with mild coronavirus disease 2019 (COVID-19) is limited.

**Methods:**

This study evaluated the association of underlying CV conditions with disease progression in patients with mild COVID-19. The primary outcome was the need to be transferred to the designated hospital for intensive care due to COVID-19 disease progression. The patients were divided into with and without CV conditions as well as stable and intensive care groups.

**Results:**

Of the 332 patients with mild COVID-19, the median age was 51 years (IQR, 40-59 years), and 200 (61.2%) were female. Of the 48 (14.5%) patients with CV conditions, 23 (47.9%) progressed to severe disease status and required intensive care. Compared with patients without CV conditions, patients with CV conditions were older and more likely to have fatigue, chest tightness, and myalgia. The rate of requiring intensive care was significantly higher among patients with CV conditions than in patients without CV conditions (47.92% vs. 12.4%; *P* < 0.001). In subgroup analysis, the rate of requiring intensive care was also higher among patients with either hypertension or coronary heart disease (CHD) than in patients without hypertension or CHD. The multivariable regression model showed that CV condition served as an independent risk factor for intensive care (odds ratio (OR), 2.652 (95% CI, 1.019-6.899)) after adjustment for various cofounders.

**Conclusions:**

Patients with mild COVID-19 complicating CV conditions are susceptible to develop severe disease status and requirement for intensive care.

## 1. Introduction

The outbreak of coronavirus disease 2019 (COVID-19), which is caused by severe acute respiratory syndrome coronavirus 2 (SARS-CoV-2) virus [1], rapidly progressed to a pandemic and was declared a public health concern [2]. Genetic sequencing of the virus suggests that SARS-CoV-2 is a betacoronavirus closely linked to the SARS virus. While most people with COVID-19 develop mild or uncomplicated illness, approximately 14% of the people develop severe disease and finally require oxygen support; unfortunately, 5% of the patients even need to be treated in the intensive care unit [3, 4]. Importantly, a large proportion of affected patients have been reported to have underlying cardiovascular diseases (CVD) [5–7] and can present additional challenge in the battle against outbreaks of novel virus infections. However, information on factors affecting disease progression among patients with mild COVID-19 is limited.

Coronaviruses are known to affect the cardiovascular system [8, 9], and the prevalence of underlying CVD is common and steadily increasing in China during recent decades, which may serve as a risk factor to the susceptibility and severity of infectious diseases [10]. Although most mild patients with COVID-19 are thought to have a favorable prognosis, those with chronic underlying conditions may suffer from worse outcomes [11]. Nevertheless, there is sparse data on clinical presentation of COVID-19 in specific populations, such as mild illness and coexisting CVD. In the present study, we report the clinical characteristics and factors associated with developing severe disease after admission in patients hospitalized into temporary hospitals [12] with mild COVID-19.

## 2. Methods

### 2.1. Study Participants and Criteria for Patient Admission, Discharge, and Referral

Consecutive patients admitted to Wuchang temporary hospital with laboratory-confirmed COVID-19 from February 5, 2020, to March 10, 2020, were included in this retrospective cohort study. The criteria for patient admission, discharge, and referral were established according to the World Health Organization interim guidance [4] and Guidance for Corona Virus Disease 2019: Prevention, Control, Diagnosis and Management (Tentative Sixth Edition) [13].

Patients who met all the following conditions were discharged alive from the hospital: (1) normal temperature for more than 3 consecutive days, (2) significantly improved respiratory symptoms, (3) resting SpO_2_ > 93%, (4) radiology examination suggesting a significantly reduced pulmonary inflammation, and (5) negative results of two consecutive SARS-CoV-2 testing by real-time reverse transcription-polymerase chain (RT-PCR) within a 24-hour interval.

Patients with one of the following conditions were referred to the designated hospital for intensive care: (1) respiratory rate ≥ 30 breaths per minute; (2) resting SpO_2_ ≤ 93%; (3) temperature ≥ 38.5°C for more than 2 consecutive days even with proper treatment; (4) severe dysfunction of the heart, liver, lung, kidney, or brain; and (5) other emergent conditions.

This study was approved by the Institutional Review Board at the Second Xiangya Hospital of Central South University and Renmin Hospital of Wuhan University. The need for written informed consent was waived by the committee.

### 2.2. Data Collection

The demographic characteristics (age and sex) and clinical data (symptoms, comorbidities, laboratory findings, and outcomes) for participants during hospitalization were collected from electronic medical records by 2 investigators. The radiologic assessments included chest radiography or computed tomography. All data were independently reviewed and entered into the computer database by 2 analysts. Cardiovascular disease (CVD) is a group of disorders of the heart and blood vessels and includes coronary heart disease (CHD), cerebrovascular disease, peripheral arterial disease, rheumatic and congenital heart diseases, and venous thromboembolism [14, 15]. According to the previous studies [16, 17], the present study includes hypertension and CVD. The patients were divided into with and without CV conditions as well as stable and intensive care groups.

### 2.3. Treatment

All patients with mild COVID-19 were treated according to the Guidance for Corona Virus Disease 2019: Prevention, Control, Diagnosis and Management (Tentative Sixth Edition). Antiviral therapy with umifenovir (0.2 g, three times daily for 5 days) or oseltamivir (75 mg, twice daily for 5 days) was administered. Traditional Chinese medicine with the Pneumonia Number 1 Decoction or Pneumonia Number 2 Decoction was also used for 3 days as a cycle until the symptoms were relieved. Statins, beta-blockers, and aspirin were administered for patients with CHD; nifedipine GITS was administered for patients with hypertension.

### 2.4. Outcome

The clinical outcomes (i.e., discharge alive and referral to the designated hospital for intensive care) were monitored up to March 25, 2020, the final date of follow-up. The primary end point was the need to be transferred to the designated hospital due to COVID-19 developing severe disease status and requirement for intensive care. Successful treatment toward hospital discharge comprised relieved clinical symptoms, normal body temperature, significant resolution of inflammation as shown by chest radiography, and at least 2 consecutive negative results shown by RT-PCR assay for SARS-CoV-2.

### 2.5. Statistical Analysis

Descriptive statistics were obtained for all study variables. All categorical variables were compared for the study outcome by using Fisher's exact test or *χ*^2^ test, and continuous variables were compared using the *t*-test or the Mann-Whitney *U* test, as appropriate. Continuous data are expressed as mean (SD) or median (interquartile range (IQR)) values. Categorical data are expressed as proportions. Survival curves were plotted using the Kaplan-Meier method and compared between patients with and without CV conditions using the log-rank test. Multivariate logistic regression models were used to determine the independent risk factors for referral during hospitalization. Data were analyzed using Stata15. Statistical charts were generated using Excel 2016 (Microsoft) or Stata15. For all the statistical analyses, *P* < 0.05 was considered significant.

## 3. Results

### 3.1. Demographics and Characteristics

**Figure 1:**
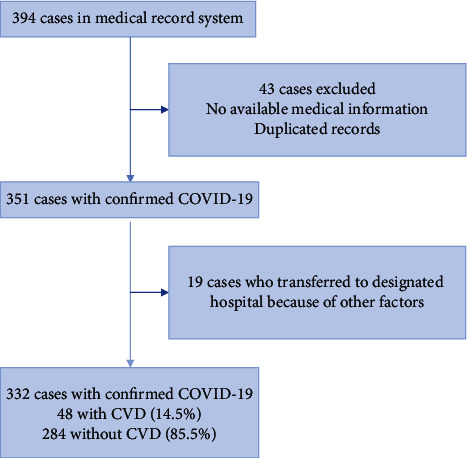
Flowchart of patient recruitment.

Figure 1 shows the flowchart for patient recruitment. Briefly, of all 394 patients in the medical record system, who were screened initially from February 5, 2020, to March 10, 2020, 43 patients without available medical information and duplicated records and 19 patients who transferred to the designated hospital because of non-COVID-19 clinical factors were excluded (8 patients requested for referral by themselves, 6 patients with anxiety and insomnia, 1 patient with manic depression, 1 patient with dysgnosia, 1 patient with chickenpox, 1 patient with acute renal colic, and 1 patient with lumbar disc herniation). Finally, the study population consisted of 332 patients hospitalized with confirmed COVID-19: 58 patients (17.5%) required referral because of requirement for intensive care (intensive group) and 274 patients (82.5%) were discharged (stable group). The median age was 51 years (median (IQR) age, IQR, 40-59 years), and 200 (61.2%) were female. Among these patients, fever (177 patients (63.2%)) was the most common symptom. Cough, fatigue, chest tightness, and myalgia were present in 108 patients (38.4%), 32 patients (11.5%), 39 patients (13.9%), and 12 patients (4.3%), respectively. Diarrhea (11 patients (3.9%)), sore throat (4 patients (1.4%)), rhinorrhea (4 patients (1.4%)), headache (7 patients (2.5%)), and toothache (2 patients (0.6%)) were rare. Hypertension (37 patients (11.1%)) and diabetes (11 patients (3.3%)) were the most common coexisting conditions.  Table 1 presents the clinical profile of stable patients and intensive care patients. Results showed that prevalence of chest tightness, toothache, CHD, hypertension, and twice or more positive SARS-CoV-2 testing was significantly higher, while positive radiology findings was significantly lower in the intensive care group than in the stable group.

Compared with patients without CV conditions, patients with CV conditions were older (56 (45-62) years vs. 50 (39-59) years; *P* = 0.007) and more likely to have fatigue (13 of 48 patients (28.3%) vs. 26 of 284 patients (11.1%); *P* = 0.002), chest tightness (18 of 48 patients (40.0%) vs. 14 of 284 patients (6.0%); *P* < 0.001), and myalgia (6 of 48 patients (13.0%) vs. 6 of 284 patients (2.6%); *P* = 0.001). Moreover, comorbidities including diabetes (4 (8.3%) vs. 7 (2.5%)) and lung disease (4 (8.3%) vs. 3 (1.1%)) were present more often among patients with CV conditions (all *P* < 0.05) (Table 2). In subgroup analysis, compared with patients without hypertension, patients with hypertension were older (56 (49-60) years vs. 50 (39-59) years; *P* = 0.009) and more likely to have fatigue (10 of 38 patients (27.0%) vs. 29 of 294 patients (11.9%); *P* = 0.014) and chest tightness (14 of 38 patients (38.9%) vs. 18 of 294 patients (7.4%); *P* < 0.001). Moreover, comorbidities including diabetes (4 (10.5%) vs. 7 (2.4%); *P* = 0.027) were present more often among patients with hypertension. Compared with patients without CHD, patients with CHD were more likely to have fatigue (5 of 11 patients (45.5%) vs. 34 of 321 patients (12.6%); *P* = 0.002) and chest tightness (6 of 11 patients (54.5%) vs. 26 of 321 patients (9.7%); *P* < 0.001). Lung disease (2 (18.2%) vs. 5 (1.6%); *P* = 0.019) was present more often among patients with CHD (Supplemental Table 1).

### 3.2. Laboratory and Radiographic Findings

In terms of laboratory findings, there were no significantly statistic differences between patients with and without CVD, including white cell count, neutrophil count, neutrophil to lymphocyte ratio (NLR), lymphocyte count, platelet count, and C-reactive protein. Results of SARS-CoV-2 testing and radiologic findings were similar between the 2 groups. There seemed to be lower radiologic abnormality observed in patients with CV conditions than those without CV conditions (52.8% vs. 67.2%) on admission. Importantly, there were more patients with progression of radiologic findings in the CV condition group than in the without CV condition group (7.1% vs. 2.6%), suggesting that the need for referral to the designated hospital for intensive care is linked to the progression of COVID-19.

### 3.3. Clinical Outcome

**Figure 2:**
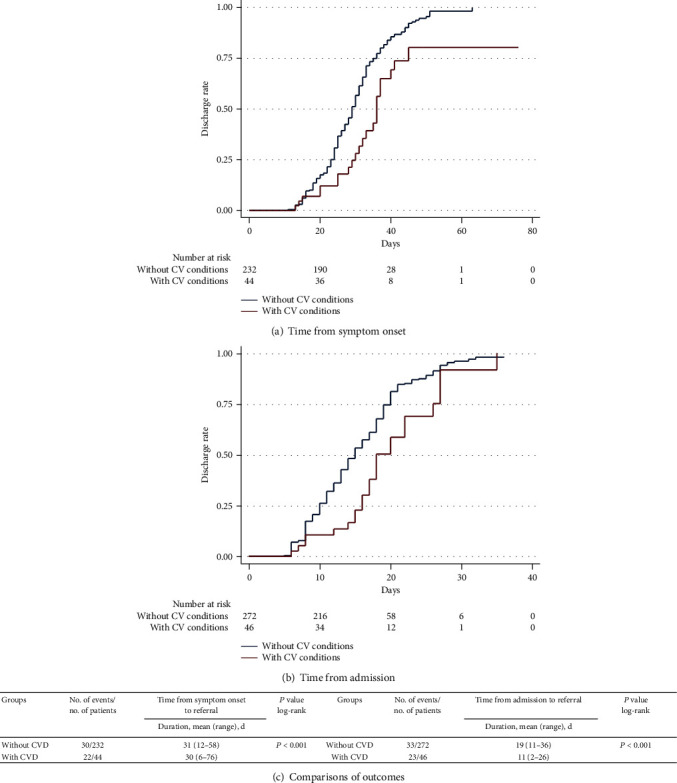
(a, b) Kaplan-Meier curves for discharge alive rate during the time from symptom onset and admission in the temporary hospital. (c) Patients with CV conditions had a lower rate of discharge alive in log-rank test, both from symptom onset and from admission. The duration means that the time from symptom onset to referral to the designated hospital for intensive care and admission in the temporary hospital to referral to the designated hospital for intensive care.

Of the 332 patients, 48 (14.5%) had underlying CV conditions including hypertension and CHD, and 23 (47.9%) developed severe diseases who required intensive care. The median time from symptom onset to admission in the temporary hospital was 12 days (IQR, 8-16 days), and the median time from admission in the temporary hospital to referral to the designated hospital was 14 days (IQR, 10-19 days). Patients with CV conditions vs. those without CV conditions had longer durations from symptom onset to admission (mean, 14 (range, 8-18) days vs. 12 (range, 8-15) days) and duration from admission to referral (15 (IQR, 8-20) days vs. 14 (IQR, 10-19) days). As shown in Table 2 and the Kaplan-Meier curves in Figure 2, the requirement for intensive care was higher among patients with vs. without CV conditions (23 of 48 (47.9%) vs. 35 of 284 (12.4%); *P* < 0.001) and discharge rate was lower in patients with CV conditions vs. without CV conditions (25 of 48 (52.1%) vs. 249 of 284 (87.6%); *P* < 0.001). In subgroup analysis, the requirement for intensive care rate was higher among patients with vs. without either hypertension or CHD and discharge rate was lower in patients with vs. without either hypertension or CHD as shown in Supplemental Table 1 and Kaplan-Meier curves in Supplemental Figures 1 and 2. As shown in Supplemental Table 2, chest tightness, platelet count, radiologic abnormality, and more than once positive SARS-CoV-2 testing by RT-PCR were risk factors of intensive care in this patient cohort. To evaluate whether CV conditions are associated with requirement for intensive care in patients with mild COVID-19, we adjusted for demographic characteristics, comorbidities, and clinical profiles: model 1 adjusted for age and sex; model 2 adjusted for age, sex, chest tightness, diabetes mellitus, and lung diseases; and model 3 adjusted for age, sex, chest tightness, diabetes mellitus, lung diseases, platelet count, radiologic abnormality, and more than once positive SARS-CoV-2 testing by RT-PCR. As shown in Table 3, CV condition remains as an independent risk factor for requirement for intensive care in patients with mild COVID-19 after adjustment for cofounders by models 1, 2, and 3.

## 4. Discussion

Since the incidence of CVD is steadily increasing in China for decades [18, 19], many hospitalized patients might naturally have comorbid CV conditions, and recent observations show that patients with coexisting CV conditions are susceptible to the most adverse complications among patients with severe COVID-19 diseases [6, 16, 20, 21, 22]. However, the impact of CV conditions on the outcome of mild COVID-19 patients is limited. The present study demonstrates that comorbid CV condition is an independent risk factor for the development of severe COVID-19 and requirement for intensive care among patients with mild COVID-19.

In the present study, among hospitalized patients with mild COVID-19, fever, cough, and fatigue were the most common symptoms, which is consistent with previous reports [14, 16]. Notably, in comparison with patients without CV conditions, patients with CV conditions showed significantly higher rates of chest tightness, fatigue, and myalgia. These finding suggests that patients with comorbidities had more severe symptoms, which clearly overlapped with the clinical symptoms of cardiovascular diseases. In the process of clinical practice, cautious screening is required to determine whether the symptoms are caused by COVID-19 or progression of underlying diseases. Particularly, chest tightness and fatigue might be associated with CV conditions including heart failure. Therefore, SARS-CoV-2 may induce and complicate the typical symptoms and manifestations of acute myocardial infarction and chronic heart failure.

Nationwide retrospective study showed that CVD was the most common coexisting condition in patients hospitalized with COVID-19 [22]. A meta-analysis of six studies including 1527 patients with COVID-19 examined the prevalence of CV conditions and reported the prevalence of hypertension, cardiac and cerebrovascular disease, and diabetes to be 17.1%, 16.4%, and 9.7%, respectively [23]. In the present study, 13.31% patients with mild cases had CV conditions. Compared to discharged patients, patients with progressive disease progression and referred to a designated hospital for intensive care were more likely to have coexisted CV conditions. Multivariate regression analysis suggested that CV condition was an independent risk factor for patients developing severe disease after adjustment of various cofounders (Table 3). In subgroup analysis, although mild COVID-19 patients with CHD had less positive radiologic findings than patients without CHD on admission, they still faced higher risk of unfavorable disease progression as compared to patients without CHD. Regarding the possible mechanisms, prevalent CHD, as an inflammation disease, may serve as a marker of accelerated immunologic dysregulation and thus aggravate the immunological imbalance induced by COVID-19. On the other hand, COVID-19 itself might aggravate the myocardial injury; recent studies from Wuhan demonstrated that severe COVID-19 patients with the most ominous outcome had significantly higher TnI or TnT levels as compared to severe COVID-19 patients undergoing recovery [24, 17]. It is known that SARS-CoV-2 can elicit the intense release of multiple cytokines and chemokines that can not only lead to vascular inflammation and plaque instability but also to myocardial inflammation [6, 19], which might be linked with the enhanced myocardial injury during the disease process. The interaction between coexisted CHD and the harm of SARS-CoV-2 on myocardial tissue might thus imply some of the mechanisms responsible for the worse outcome among mild COVID-19 patients hospitalized.

Among patients with mild cases, hypertension was the most common comorbidity, followed by CHD. An early analysis of underlying clinical features in 41 COVID-19 fatal cases revealed the highest prevalence of hypertension (60.9%) [6]. Higher expression of ACE2 in patients with hypertension has been postulated to enhance susceptibility to SARS-CoV2 [25], although the data are conflicting and without clear suggestion implication for treatment [26]. Additional study is needed to understand the potential mechanistic relationships between hypertension and COVID-19 outcomes.

## 5. Limitations

Our study has several limitations. First, restricted by the limited medical resources, laboratory biochemical testing was insufficient; second, regarding the time of disease onset in patients, the uncertainty of the exact dates (recall bias) might have inevitably affected our assessment; lastly, data from the earliest hospitalized patients could not be fully accessed.

## 6. Clinical Implication

Mild COVID-19 patients with coexisting CV conditions should receive more intensive monitoring to prevent the transition to severe disease progression.

## 7. Conclusions

Although most mild COVID-19 patients could be discharged, approximately half of the mild COVID-19 patients with comorbid CV conditions may progress to severe disease status and require intensive care; special monitoring on these patient populations is essential to improve the outcome of these patients.

## Figures and Tables

**Table 1:** Clinical features of patients with mild COVID-19.

Characteristic	All patients (*n* = 332)	Stable group (*n* = 274, 82.5%)	Intensive care group (*n* = 58, 17.5%)	*P* value
*Sex*				
Male, *n* (%)	132 (39.8)	105 (38.3)	27 (46.6)	0.245
Female, *n* (%)	200 (61.2)	169 (61.7)	31 (53.4)	0.245
*Age, median (IQR)*	51 (40-59)	50.5 (40-59)	52 (42-60)	0.478

*Symptoms*				
Fever, *n* (%)	177 (63.2)	142 (63.4)	35 (62.5)	0.901
Cough, *n* (%)	108 (38.4)	88 (39.1)	20 (35.7)	0.640
Fatigue, *n* (%)	39 (13.9)	28 (12.5)	11 (19.6)	0.167
Chest tightness, *n* (%)	32 (11.5)	15 (6.7)	17 (29.3)	<0.001
Diarrhea, *n* (%)	11 (3.9)	8 (3.6)	3 (5.4)	0.538
Headache, *n* (%)	7 (2.5)	6 (2.7)	1 (1.8)	0.702
Sore throat, *n* (%)	4 (1.4)	2 (0.9)	2 (3.4)	0.181
Rhinorrhea, *n* (%)	4 (1.4)	4 (1.8)	0 (0.0)	0.587
Myalgia, *n* (%)	12 (4.3)	9 (4.0)	3 (5.4)	0.658
Toothache, *n* (%)	2 (0.6)	0 (0.0)	2 (3.4)	0.030

*Comorbidities*				
CV conditions, *n* (%)	48 (14.5)	25 (7.5)	23 (39.7)	<0.001
CHD, *n* (%)	11 (3.3)	1 (0.4)	10 (17.2)	<0.001
Hypertension, *n* (%)	38 (11.4)	21 (7.7)	17 (27.9)	<0.001
Diabetes, *n* (%)	11 (3.3)	7 (2.6)	4 (6.9)	0.106
Lung disease, *n* (%)	7 (2.1)	4 (1.5)	3 (5.2)	0.105
*Radiologic abnormality,n(%)*	201 (65.5)	183 (70.1)	18 (36.7)	<0.001

*Positive SARS-CoV-2 testing by RT-PCR*				
Twice or more, *n* (%)	72 (23.0)	48 (18.3)	24 (48.0)	<0.001
Once, *n* (%)	241 (77.0)	215 (81.7)	26 (74)	<0.001

*Laboratory findings*				
Red blood cell count (∗10^12^/L), median (IQR)	4.39 (4.05-4.73)	4.39 (4.05-4.73)	4.42 (4.02-4.76)	0.863
White cell count (∗10^9^/L), median (IQR)	5.25 (4.49-6.37)	5.19 (4.47-6.33)	5.62 (4.88-6.58)	0.058
Neutrophil count (∗10^9^/L), median (IQR)	3.21 (2.57-4.08)	3.18 (2.55-3.98)	3.49 (2.67-4.27)	0.143
Lymphocyte count (∗10^9^/L), median (IQR)	1.61 (1.26-1.91)	1.58 (1.26-1.86)	1.80 (1.26-2.17)	0.057
NLR, median (IQR)	2.1 (1.6-2.7)	2.1 (1.6-2.7)	2.1 (1.5-2.8)	0.911
Platelet count (∗10^9^/L), median (IQR)	258 (216-310)	267 (218-324)	223 (194-269)	0.003
Hemoglobin (g/L), median (IQR)	135 (125-145)	135 (126-144)	135 (122-145)	0.781

C-reactive protein				
<10.00 mg/L	261 (85.0)	217 (84.4)	44 (88.0)	0.518
≥10.00 mg/L	46 (15.0)	40 (15.6)	6 (12.0)	0.518
*Median length of symptoms onset to admission (days) (IQR)*	12 (8-16)	12 (8-15)	13 (6-21)	0.679
*Median length of temporary hospital stay (days) (IQR)*	14 (10-19)	14 (10-19)	15 (11-21.5)	0.322

IQR: interquartile range; NLR: neutrophil to lymphocyte ratio; CV: cardiovascular; CHD: coronary heart disease.

**Table 2:** Clinical characteristics of patients with COVID-19 with or without CV conditions.

Characteristic	All patients (*n* = 332)	Without CV conditions (*n* = 284, 85.5%)	With CV conditions (*n* = 48, 14.5%)	*P* value
*Sex*				
Male, *n* (%)	132 (39.8)	110 (38.7)	22 (45.8)	0.285
Female, *n* (%)	200 (60.2)	174 (61.3)	26 (54.2)	0.285
*Age, median (IQR)*	51 (40-59)	50 (39-59)	56 (45-62)	0.007

*Symptoms*				
Fever, *n* (%)	177 (63.2)	150 (63.3)	27 (58.7)	0.487
Cough, *n* (%)	108 (38.4)	88 (37.6)	20 (42.6)	0.525
Fatigue, *n* (%)	39 (13.9)	26 (11.1)	13 (28.3)	0.002
Chest tightness, *n* (%)	32 (11.5)	14 (6.0)	18 (40)	<0.001
Diarrhea, *n* (%)	11 (3.9)	9 (3.8)	2 (4.3)	0.873
Headache, *n* (%)	7 (2.5)	5 (2.1)	2 (4.3)	0.323
Sore throat, *n* (%)	4 (1.4)	4 (1.7)	0 (0.0)	1.000
Rhinorrhea, *n* (%)	4 (1.4)	4 (1.7)	0 (0.0)	1.000
Myalgia, *n* (%)	12 (4.3)	6 (2.6)	6 (13.0)	0.001
Toothache, *n* (%)	2 (0.6)	1 (0.4)	1 (2.1)	0.269

*Comorbidities*				
Diabetes, *n* (%)	11 (3.3)	7 (2.5)	4 (8.3)	0.036
Lung disease, *n* (%)	7 (2.1)	3 (1.1)	4 (8.3)	0.010
*Radiologic abnormality,n(%)*	201 (65.5)	182 (67.2)	19 (52.8)	0.088

*Positive SARS-CoV-2 testing by RT-PCR*				
Twice or more, *n* (%)	72 (23.0)	60 (22.0)	12 (35.1)	0.147
Once, *n* (%)	241 (77.0)	216 (78.0)	25 (64.9)	0.147

*Laboratory findings*				
Red blood cell count (∗10^12^/L), median (IQR)	4.39 (4.05-4.73)	4.37 (4.04-4.68)	4.49 (4.15-4.90)	0.159
White cell count (∗10^9^/L), median (IQR)	5.25 (4.49-6.37)	5.25 (4.49-6.37)	5.00 (4.46-6.35)	0.677
Neutrophil count (∗10^9^/L), median (IQR)	3.21 (2.57-4.08)	3.23 (2.59-4.10)	3.06 (2.53-3.91)	0.468
Lymphocyte count (∗10^9^/L), median (IQR)	1.61 (1.26-1.91)	1.58 (1.27-1.91)	1.72 (1.23-1.92)	0.745
NLR, median (IQR)	2.06 (1.60-2.67)	2.07 (1.60-2.67)	2.04 (1.56-2.54)	0.609
Platelet count (∗10^9^/L), median (IQR)	258 (216-310)	263 (217-314)	243 (202-287)	0.085
Hemoglobin (g/L), median (IQR)	135 (125-145)	135 (125-145)	137 (130-145)	0.254

C-reactive protein				
<10.00 mg/L	261 (85.0)	230 (85.8)	31 (79.5)	0.300
≥10.00 mg/L	46 (15.0)	38 (14.2)	8 (20.5)	0.300
*Median length of symptom onset to admission (days) (IQR)*	12 (8-16)	12 (8-15)	14 (8-18)	0.222
*Median length of temporary hospital stay (days) (IQR)*	14 (10-19)	14 (10-19)	15 (8-20)	0.837

*Clinical outcomes*				
Discharge, *n* (%)	274 (82.5)	249 (87.6)	25 (52.1)	<0.001
Require intensive care, *n* (%)	58 (17.5)	35 (12.4)	23 (47.9)	<0.001

IQR: interquartile range; NLR: neutrophil to lymphocyte ratio.

**Table 3:** Multivariable analyses of the risk factors for intensive care in patients with mild COVID-19.

	Unadjusted			Model 1			Model 2			Model 3		
	OR	95% CI	*P*	OR	95% CI	*P*	OR	95% CI	*P*	OR	95% CI	*P*
With CV conditions	6.545	3.357-12.762	0.001	6.430	3.210-12.878	<0.001	3.941	1.798-8.636	0.001	2.652	1.019-6.899	0.046
Age	1.006	0.983-1.030	0.604	0.994	0.969-1.020	0.647	0.993	0.966-1.020	0.601	0.991	0.961-1.021	0.545
Sex	1.402	0.792-2.480	0.246	1.284	0.699-2.359	0.007	1.384	0.720-2.663	0.330	0.960	0.447-2.062	0.916
Chest tightness	6.044	2.788-13.107	0.001				3.451	1.422-8.376	0.006	3.340	1.070-10.429	0.038
Diabetes mellitus	2.825	0.799-9.987	0.107				2.473	0.606-10.087	0.207	2.784	0.604-12.826	0.189
Lung diseases	3.682	0.801-16.915	0.094				3.604	0.557-23.327	0.178	3.806	0.480-30.183	0.206
Platelet	0.991	0.986-0.996	<0.001							0.233	0.075-0.726	0.012
Radiologic abnormality	0.238	0.125-0.451	0.001							0.218	0.101-0.468	0.001
Positive SARS-CoV-2 testing by RT-PCR ≥ 2	4.135	2.187-7.817	0.001							3.754	1.719-8.197	0.001

Sample size: *n* = 332. Data are expressed as OR ± 95%CIs (reported in parentheses) as assessed by univariate (unadjusted) or multivariate logistic regression analyses. OR: odds ratio; CI: confidence interval; CV: cardiovascular. In these logistic regression models, CV condition was a categorical variable. Other covariates included in multivariate logistic regression models were as follows: model 1: adjustment for age and sex; model 2: adjustment for variables including age, sex, chest tightness, diabetes mellitus, and lung diseases; model 3: adjustment for variables including age, sex, chest tightness, diabetes mellitus, lung diseases, platelet, radiologic abnormality, and positive SARS-CoV-2 testing by RT-PCR.

## Data Availability

The data used to support the findings of this study are included within the supplementary information file.

## Additional Points

*Key Points. Question*. What is the impact of coexisting cardiovascular (CV) diseases on disease progression in patients with mild COVID-19? *Findings*. Although most patients with mild COVID-19 were discharged alive from the hospital, approximately 47.9% patients with coexisting CV conditions developed severe disease status and required intensive care. CV condition is an independent risk factor of intensive care among patients with mild COVID-19. *Meaning*. Coexisting CV conditions are associated with unfavorable outcomes among patients with mild COVID-19. Special monitoring is required for these patients to improve their outcome.

## Supplementary Materials

Supplementary Materials Supplemental Table 1: subgroup analysis of patients with and without hypertension or CHD. Supplemental Table 2: univariable analyses of the risk factors between the stable group and the intensive group. Supplemental Figure 1: discharge rate between patients with and without hypertension (HTN). Supplemental Figure 2: discharge rate between patients with and without CHD.
