# Supplementary material for: Association between Cardiovascular Burden and Requirement of Intensive Care among Patients with Mild COVID-19
Source: Cardiovasc Ther. 2020 Aug 1;2020:9059562. doi: 10.1155/2020/9059562 (PMC7436340; doi:10.1155/2020/9059562)
Supplement: Supplementary Materials — Supplemental Table 1: subgroup analysis of patients with and without hypertension or CHD. Supplemental Table 2: univariable analyses of the risk factors between the stable group and the intensive group. Supplemental Figure 1: discharge rate between patients with and without hypertension (HTN). Supplemental Figure 2: discharge rate between patients with and without CHD. [file 9059562.f1.pdf]

Supplemental Table 1. Subgroup analysis of patients with and without hypertension or CHD

	Hypertension		*P* value	CHD		*P* value
	Without (n=294，88.6%)	With (n=38，11.4%)		Without (n=321，96.7%)	With (n=11，3.3%)	
**Sex**						
Male，n（%）	112(38.1)	20(52.6)	0.085	127（39.6）	5（45.5）	0.759
Female，n（%）	182(61.9)	18(47.4)	0.085	194（60.4）	6（54.5）	0.759
**Age, median (IQR)**	50（39-59）	56（49-60）	0.009	51(40-59)	56(45-64)	0.174
**Symptoms**						
Fever，n（%）	156(64.2)	21(56.8)	0.382	169（62.8）	8（72.7）	0.751
Cough，n（%）	93(38.3)	15(39.5)	0.887	104(32.4)	4(36.4)	0.885
Fatigue，n（%）	29(11.9)	10(27.0)	0.014	34(12.6)	5(45.5)	0.002
Chest Tightness，n（%）	18(7.4)	14(38.9)	<0.001	26(9.7)	6(54.5)	<0.001
Diarrhea，n（%）	9(3.7)	2(5.4)	0.644	10(3.1)	1(9.1)	0.362
Headache，n（%）	5(2.1)	2(5.4)	0.233	7(2.6)	0(0.0)	1.000
Sore throat，n（%）	4(1.7)	0(0.0)	1.000	4(1.5)	0(0.0)	1.000
Rhinorrhea，n（%）	4(1.7)	0(0.0)	1.000	4(1.5)	0(0.0)	1.000
Myalgia，n（%）	9(3.7)	3(8.1)	0.233	11(4.1)	1(9.1)	0.388
Toothache，n（%）	2（0.7）	0（0.0）	1.000	1(0.3)	1(9.1)	0.065
**Comorbidities**						
Diabetes，n（%）	7(2.4)	4(10.5)	0.027	11(34.3	0(0.0)	0.532
Lung disease，n（%）	5(1.7)	2(5.3)	0.185	5(1.6)	2(18.2)	0.019
**Radiologic abnormality，n（%）**	185（66.3）	16（57.1）	0.331	199(66.6)	2(25.0)	0.022

**Positive SARS-CoV-2 testing by RT-PCR**						
Twice or more, n (%)	64(22.5)	8(28.6)	0.463	69（22.6）	3（37.5）	0.391
Once, n（%）	221(77.5)	20(71.4)	0.463	236（77.4）	5（62.5）	0.391
**Laboratory findings**						
Red-blood cell count*10 12/L, median (IQR)	4.37（4.02-4.69）	4.51（4.16-4.90）	0.094	4.39（4.05-4.74）	4.28（4.00-4.54）	0.506
White-cell count *10 9/L, median (IQR)	5.25（4.48-6.37）	5.55（4.74-6.35）	0.875	5.25（4.49-6.37）	5.19（4.60-5.93）	0.816
Neutrophil count *10 9/L, median (IQR)	3.21（2.58-4.12）	3.23（2.55-3.80）	0.828	3.24（2.58-4.08）	2.85（2.25-3.69）	0.392
Lymphocyte count*10 9/L, median (IQR)	1.58（1.26-1.91）	1.73（1.26-1.92）	0.397	1.61（1.26-1.91）	1.58(1.20-2.39)	0.683
NLR, median (IQR)	2.07（1.57-2.69）	2.04（1.65-2.37）	0.729	2.07（1.61-2.67）	1.66(1.11-3.06)	0.340
Platelet count*10 9/L, median (IQR)	261（216-313）	255（204-289）	0.325	259（216-312）	221(193-270)	0.254
Hemoglobin, g/L, median (IQR)	135（125-144）	139（131-146）	0.154	135（125-145）	135(125-143)	0.869
C-reactive protein,						
<10.00 mg/L	236(85.2)	25(83.3)	0.788	255(85.3)	6(75.0)	0.421
≥10.00 mg/L	41(14.8)	5(16.7)	0.788	44(14.7)	2(25.0)	0.421
**Median length of symptoms onset to admission，days**	12（7-17）	13（7-17）	0.942	12(8-16)	17(7-26)	0.131

**（IQR）**						
**Median length of temporary hospital stay，days （IQR）**	14（10-19）	15（7-20）	0.730	14(10-19)	15(2-18)	0.487
**Clinical outcomes**						
Discharge, n（%）	253（86.1）	21（55.3）	0.001	273(85.0)	1(9.1)	0.001
Require intensive care, n （%）	41（13.9）	17（44.7）	0.001	48(15.0)	10(90.9)	0.001

Abbreviation: CHD: coronary heart disease; IQR, interquartile range; NLR: neutrophil to lymphocyte ratio.

Supplemental Table 2. Univariable analyses of the risk factors between stable group and intensive group

Characteristic	Stable group (n=274，82.5%)	Intensive group (n=58，17.5%)	*β*	*OR*	*CI*	*P* Value
**Sex**						
Male，n（%）	105（38.3）	27（46.6）	0.337	1.402	0.792-2.480	0.246
**Age, median (IQR)**	50.5（40-59）	52（42-60）	0.006	1.006	0.983-1.030	0.604
**Symptoms**						
Fever，n（%）	142（63.4）	35（62.5）	-0.038	0.962	0.525-1.763	0.901
Cough，n（%）	88（39.1）	20（35.7）	-0.145	0.865	0.471-1.590	0.640
Fatigue，n（%）	28（12.5）	11（19.6）	0.537	1.711	0.793-3.692	0.171
Chest Tightness，n（%）	15（6.7）	17（29.3）	1.799	6.044	2.788-13.107	<0.001
Diarrhea，n（%）	8（3.6）	3（5.4）	0.424	1.528	0.392-5.957	0.541
Headache，n（%）	6(2.7)	1(1.8)	-0.415	0.661	0.078-5.601	0.704
Sore throat，n（%）	2(0.9)	2(3.4)	1.409	4.093	0.564-29.713	0.164
Rhinorrhea，n（%）	4(1.8)	0(0.0)	0	1		
Myalgia，n（%）	9(4.0)	3(5.4)	0.302	1.352	0.354-5.168	0.659
**Comorbidities**						
CV conditions，n（%）	25(7.5)	23(39.7)	1.879	6.545	3.357-12.762	<0.001
CHD，n（%）	1(0.4)	10(17.2)	4.040	56.875	7.117-454.518	<0.001
Hypertension，n（%）	21（7.7）	16（27.9）	1.609	4.995	2.433-10.258	<0.001
Diabetes，n（%）	7（2.6）	4（6.9）	1.039	2.825	0.799-9.987	0.107
Lung disease，n（%）	4（1.5）	3（5.2）	1.303	3.682	0.801-16.915	0.094
**Radiologic abnormality，n（%）**	183（70.1）	18（36.7）	-1.436	0.238	0.125-0.451	<0.001
**Positive SARS-CoV-2 testing by RT-PCR**						
Twice or more，n（%）	48（18.3）	24（48.0）	1.419	4.135	2.187-7.817	<0.001

Once，n（%)	215（81.7）	26（74）	1.419	4.135	2.187-7.817	<0.001
**Laboratory findings**						
Red-blood cell count*10[12]/L, median (IQR)	4.39(4.05-4.73)	4.42(4.02-4.76)	-0.959	0.909	0.532-1.552	0.726
White-cell count *10[9]/L, median (IQR)	5.19(4.47-6.33)	5.62(4.88-6.58)	0.192	1.212	1.010-1.453	0.058
Neutrophil count *10[9]/L, median (IQR)	3.18(2.55-3.98)	3.49(2.67-4.27)	0.191	1.211	0.957-1.532	0.111
Lymphocyte count, *10[9]/L, median (IQR)	1.58(1.26-1.86)	1.80(1.26-2.17)	0.634	1.886	1.073-3.314	0.027[#]
NLR, median (IQR)	2.1（1.6-2.7）	2.1（1.5-2.8）	-0.027	0.973	0.716-1.324	0.863
Platelet count*10[9]/L, median (IQR)	267(218-324)	223(194-269)	-0.009	0.991	0.987-0.996	0.001
Hemoglobin, g/L, median (IQR)	135(126-144)	135(122-145)	-0.006	0.994	0.977-1.011	0.497
C-reactive protein						
≥10.00 mg/L	40（15.6）	6（12.0）	-0.301	0.740	0.296-1.851	0.520
**Median length of symptoms onset to admission，days （IQR）**	12（8-15）	13（6-21）	0.025	1.025	0.991-1.061	0.157
**Median length of temporary hospital stay，days （IQR）**	14（10-19）	15（11-21.5）	0.023	1.024	0.979-1.070	0.303

Abbreviation: IQR, interquartile range; NLR: neutrophil to lymphocyte ratio; CHD: coronary heart disease.

Supplemental Figure 1 Discharge rate between patients with vs without hypertension (HTN)

A. Time from symptom onset

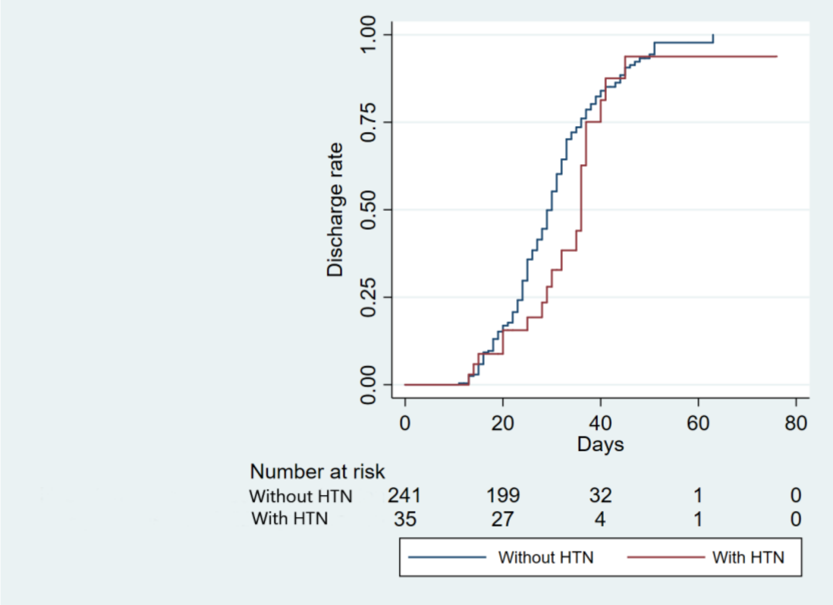

B. Time from admission

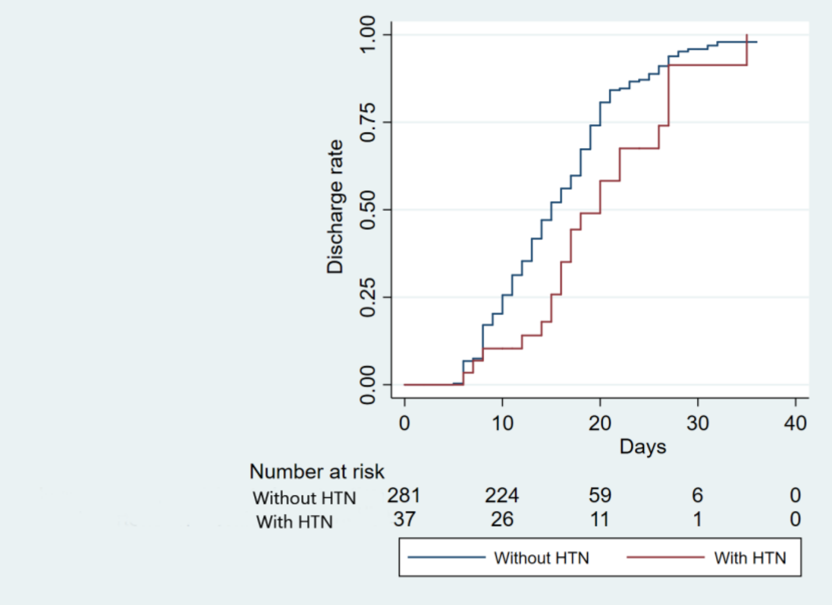

C. Comparisons of outcomes

Groups	No. of events/ No. of patients	Time from symptom onset to referral<br>Duration, Mean (range), d	P value log-rank	Groups	No. of events/ No. of patients	Time from admission to referral<br>Duration, Mean (range), d	P value log-rank
Without hypertension	36/241	33(12-58)	P<0.001	Without hypertension	39/281	18(11-36)	P<0.001
With hypertension	16/35	26(6-76)		With hypertension	17/37	10(2-26)	

Supplemental Figure 2 Discharge rate between patients with vs without CHD

A. Time from symptom onset

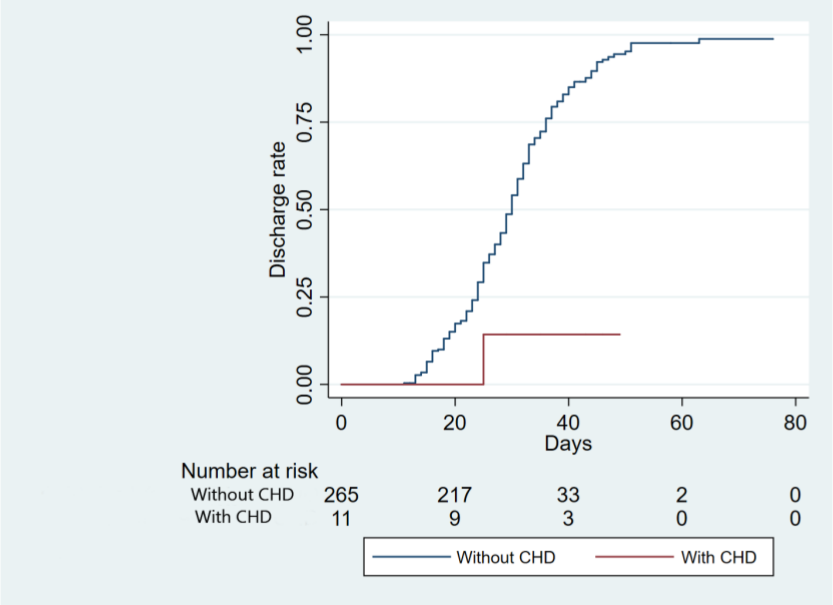

B. Time from admission

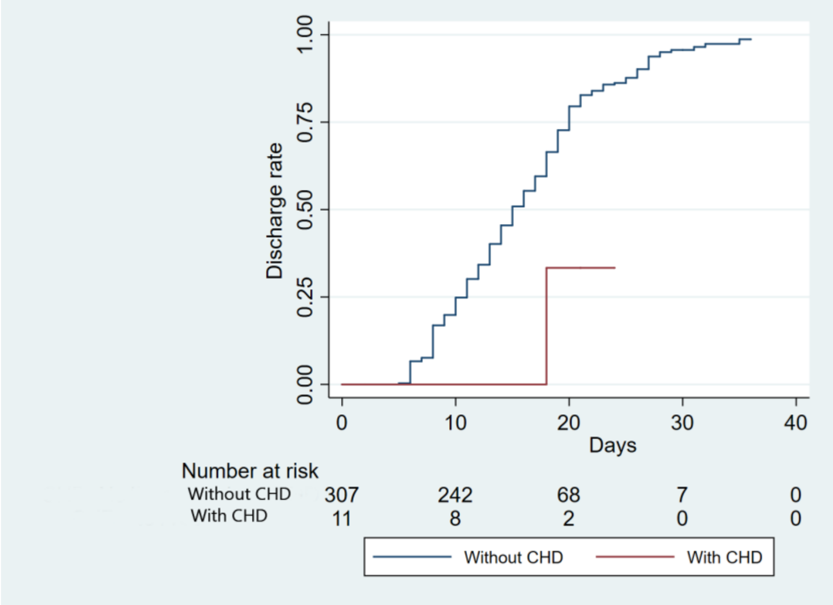

C. Comparisons of outcomes

Groups	No. of events/ No. of patients	Time from symptom onset to referral<br>Duration, Mean (range), d	P value log-rank	Groups	No. of events/ No. of patients	Time from admission to referral<br>Duration, Mean (range), d	P value log-rank
Without CHD	42/265	31(6-76)	P<0.001	Without CHD	46/307	17(2-36)	P<0.001
With CHD	10/11	31(17-49)		With CHD	10/11	12(2-24)	
